# Anatomic Pathways of Peripancreatic Fluid Draining to Mediastinum in Recurrent Acute Pancreatitis: Visible Human Project and CT Study

**DOI:** 10.1371/journal.pone.0062025

**Published:** 2013-04-17

**Authors:** Haotong Xu, Xiaoming Zhang, Andreas Christe, Lukas Ebner, Shaoxiang Zhang, Zhulin Luo, Yi Wu, Yin Li, Fuzhou Tian

**Affiliations:** 1 Postdoctoral Workstation, the General Surgery Center of the Peoples’ Liberation Army, Chengdu Army General Hospital, Chengdu, Sichuan, P. R. China; 2 Department of Radiology, Second Affiliated Hospital, North Sichuan Medical College, Nanchong, Sichuan, P. R. China; 3 Sichuan Key Laboratory of Medical Imaging, Department of Radiology, Affiliated Hospital of North Sichuan Medical College, Nanchong, Sichuan, P. R. China; 4 Department of Radiology, Inselspital, University of Bern, Bern, Switzerland; 5 Department of Anatomy, College of Basic Medical Sciences, Third Military Medical University, Chongqing, P. R. China; University of Washington School of Medicine, United States of America

## Abstract

**Background:**

In past reports, researchers have seldom attached importance to achievements in transforming digital anatomy to radiological diagnosis. However, investigators have been able to illustrate communication relationships in the retroperitoneal space by drawing potential routes in computerized tomography (CT) images or a virtual anatomical atlas. We established a new imaging anatomy research method for comparisons of the communication relationships of the retroperitoneal space in combination with the Visible Human Project and CT images. Specifically, the anatomic pathways of peripancreatic fluid extension to the mediastinum that may potentially transform into fistulas were studied.

**Methods:**

We explored potential pathways to the mediastinum based on American and Chinese Visible Human Project datasets. These drainage pathways to the mediastinum were confirmed or corrected in CT images of 51 patients with recurrent acute pancreatitis in 2011. We also investigated whether additional routes to the mediastinum were displayed in CT images that were not in Visible Human Project images.

**Principal Findings:**

All hypothesized routes to the mediastinum displayed in Visible Human Project images, except for routes from the retromesenteric plane to the bilateral retrorenal plane across the bilateral fascial trifurcation and further to the retrocrural space via the aortic hiatus, were confirmed in CT images. In addition, route 13 via the narrow space between the left costal and crural diaphragm into the retrocrural space was demonstrated for the first time in CT images.

**Conclusion:**

This type of exploration model related to imaging anatomy may be used to support research on the communication relationships of abdominal spaces, mediastinal spaces, cervical fascial spaces and other areas of the body.

## Introduction

In recent years, researchers have focused on how they could transform the scientific findings of molecular biology and neurobiology to clinical treatments [1], [2]. However, they have seldom paid attention to achievements in transforming digital anatomy to radiological diagnosis. Because the images in the Visible Human Project datasets form high resolution images of human specimens, they are far clearer than those derived from computerized tomography (CT) and magnetic resonance imaging (MRI) [3]. The Visible Human Project, which is based on thin-slice cross-sectional human datasets, may provide a platform for transforming research findings in digital anatomy into higher accuracy in clinical diagnosis and new surgical approaches and operative patterns [3], [4].

In previous studies on communication relations in the retroperitoneal space and the pelvic extraperitoneal space, investigators illustrated these communication relationships by drawing potential routes in CT images or on a computer-generated virtual anatomical atlas [5], [6]. Confined by the spatial resolution of these images or imaging modality to the internal structures of the human body, images of the fascial planes, ligaments and spaces outside the viscera could not be manifested satisfactorily. However, the fascial planes, ligaments, spatial relationships between fascial planes and adjacent retroperitoneal spaces can be presented with high resolution in Visible Human Project images [7]. In addition, the Visible Human Project images can restore the original, undisrupted anatomical relations of body regions in patient images [3]. The pancreatic fluid extends along fascial planes and bridges the septa in acute pancreatitis. The communication relationships of the retroperitoneal space may be reflected with objective insight in CT images [8]. Therefore, it is promising to explore the communication relationships of the retroperitoneal space with Visible Human Project and CT images in conjunction.

Molmenti and Aizenstein firstly proposed a new concept of fascial planes [9], [10]. As the preferential drainage paths, the fascial planes and perirenal bridging septa can be recruited to rapidly drain developing fluid collections. There has been agreement on the spatial relationship between the left fascial plane upper end points and the left extraperitoneal space inferior to the esophageal hiatus as well as the relationship between the right fascial plane upper end points and the hepatic bare area below the inferior vena cava hiatus [5], [7]. Moreover, the relationships among the retromesenteric plane, retrorenal plane and opening of the aortic hiatus have been clarified [8], [11]. Based on the above anatomical relationships, the pathways of fluid drainage to the mediastinum were explored.

Traditionally, large amounts of pancreatic fluid will spread along preferential drainage pathways into the posterior pararenal space and pelvic extraperitoneal space in acute pancreatitis [5], [9], [11]. Unusual drainage pathways may extend to the mediastinum. In most of the reported cases of mediastinal extension of pancreatic fluid collections, patients had histories of: (1) previous upper abdominal trauma or surgery; (2) alcoholism; or (3) previous hospitalization for pancreatitis. Because the more common pathways for pancreatic fluid offer greater resistance due to fibrosis from previous inflammation, the spread of fluid occurs along the path of least resistance [12]. In this case, mediastinal extension of pancreatic fluid may occur. Ball and Mallavarapu et al. carried out a statistical analysis on which diaphragmatic hiatus the fluid would enter into when draining to the mediastinum in acute pancreatitis [12], [13]. However, they did not consider the detailed anatomic pathways of the peripancreatic fluid draining into the mediastinum. With pathological progression of acute pancreatitis, the above transdiaphragmatic fluid extension then forms a mediastinal pseudocyst or pancreaticopleural fistula. These two entities can produce large and recurrent pleural effusions [14], [15]. Furthermore, they can result in an increase in morbidity and mortality [16].

As rare complications, the presentation of these two diseases is often confusing [15], [17]. Perhaps the pathological anatomy is not fully understood, and they are misdiagnosed or missed. Therefore, our study established a new pattern for exploring the communication relationships of the retroperitoneal space in combination with Visible Human Project and CT images. Specifically, the pathways of the peripancreatic fluid extension to the mediastinum were explored to provide an anatomic basis for identifying mediastinal pseudocysts and pancreaticopleural fistulas.

## Materials and Methods

### 1 Ethics statement

The study was approved by the Ethics Review Board of the Chengdu Army General Hospital. The First Chinese Visible Human (CVH1), Second Chinese Visible Human (CVH2) and Fifth Chinese Visible Human (CVH5) datasets of the Chinese Visible Human Project (CVHP) were voluntarily donated [3]. Written consent was obtained from relatives of the participants in CVH1, CVH2 and CVH5. Obtaining CT scans in 51 patients with recurrent acute pancreatitis was approved by the Institutional Review Board of the Chengdu Army General Hospital. Written informed consent was obtained from the 51 patients before CT scans, and no identifiable information (i.e., age and gender) was reported in this study.

### 2 Experimental design overview

In this new imaging anatomy research method for comparative study of detailed communication relationships among retroperitoneal spaces, the experimental pipeline consisted of three stages: exploring potential pathways to the mediastinum based on Visible Human Project datasets; confirming and correcting drainage pathways to the mediastinum in CT images; discovering new conduits to the mediastinum in CT images and seeking anatomic evidence from Visible Human Project images.

### 3 Observation of pathways in Visible Human datasets

The successive thin-slice cross-sectional images of the upper and middle abdomen, from the retrocrural space to the combined interfascial planes, including the slices of drainage pathways, were retrieved from the CVHP datasets. The axial slice interval of the CVHP was 0.2 mm or 0.5 mm and the resolution was 6,291,456 (3,072×2,048) pixels, as described previously [3]. In addition to the CVHP datasets, the dataset for the American Visible Human Project (VHPA) was obtained from the US National Library of Medicine [4].The Virtual Human dataset of Norman Bethune Medical College (VHBM) was provided by Jilin University [18]. The cadavers underwent preliminary CT and MRI scans to exclude lesions of the abdomen before milling. We analyzed potential routes into the mediastinum via the diaphragmatic hiatuses on Visible Human Project axial images by considering the concept of fascial planes [9], [10]. For the esophageal hiatus and inferior vena cava hiatus at the midriff top, the potential pathways to the mediastinum across them were presented on reconstructed oblique sagittal 3D sections based on 450 serial cross-sectional images from the CVH2 dataset using multiply reconstructed (MPR) technology with Amira® software. The Amira® software, which is one of the medical 3D reconstruction software programs, has the advantage of showing colorful images of Visible Humans Project as well as CT images for random sections.

### 4 Patients

Medical records and CT images were reviewed retrospectively for consecutive patients with recurrent acute pancreatitis admitted to our institute between January 2011 and November 2011. Recurrent acute pancreatitis was defined as two or more separate attacks of pancreatitis. All patients that had accompanying mediastinal extension of peripancreatic fluid collections were included in our research. Patients were excluded with primary acute pancreatitis diagnosed by the International Classification of Diseases, Ninth Revision, Clinical Modification code for acute pancreatitis (577.0) [19]. Patients with chronic pancreatitis, based on history, CT scan, abdominal ultrasound, or prior post-endoscopic retrograde cholangiopancreatography, were also excluded. Thus, a total of 51 patients were recruited as our research subjects.

### 5 Abdominal CT techniques

All patients underwent abdominal CT scans in the Chengdu Army General Hospital. CT scans was performed on a 64-slice CT scanner (Siemens Medical Solutions, Erlangen, Germany). CT parameters were as follows: 120 kVp, 220–260 mAs, 1.5-mm beam collimation, 5-mm section thickness, 330 ms rotation time and a standard reconstruction algorithm. One hundred and fifty milliliters of iodine contrast material (Iopamiron 300, Schering, Berlin, Germany) were administered intravenously to 40 patients at a flow rate of 3–5 ml/s. The area scanned extended from the retrocrural space to the iliac crest.

### 6 Observation of pathways in CT images

Firstly, all CT examinations that were obtained 3–5 days after admission were reviewed by two abdominal radiologists (each with at least ten years of experience in interpreting abdominal CT images) without knowledge of the patient’s clinical course. They recorded a consensus evaluation of CT findings including the diagnosis of recurrent acute pancreatitis and the pathways of the peripancreatic fluid to the mediastinum in CT images. Drainage pathways to the mediastinum were evaluated based on our own predefined criteria. In normal conditions, the retroperitoneal space and the mediastinal space contained only connective or fat tissue. The fascial planes could not be visualized or were only present as thin, white line in CT scans of healthy people [20]. When drainage pathways to the mediastinum were composed, the typical CT appearance was fluid distributing along dissected fascial planes and adjacent spaces to the mediastinum [5], [21]. The aforementioned spaces were present with haziness and streaky density with or without fluid collections [22]. Secondly, the routes to the mediastium across the esophageal hiatus and inferior vena cava hiatus were observed on reformed sagittal and oblique sagittal sections in some patients corresponding to the pathways displayed on CVH2 reconstructed images with Amira® software. The reformed thickness and interval of the MPR images was 0.33 mm. Lastly, we looked for differences between drainage pathways in CT images compared to Visible Human images.

### 7 Exploring additional pathways in CT vs. CVH2 images

If some additional conduits to the mediastinum were manifested while observing routes via the diaphragmatic hiatuses into the mediastinum in CT images, we considered the anatomic evidence that formed these conduits in Visible Human Project images.

## Results

**Figure 1:**
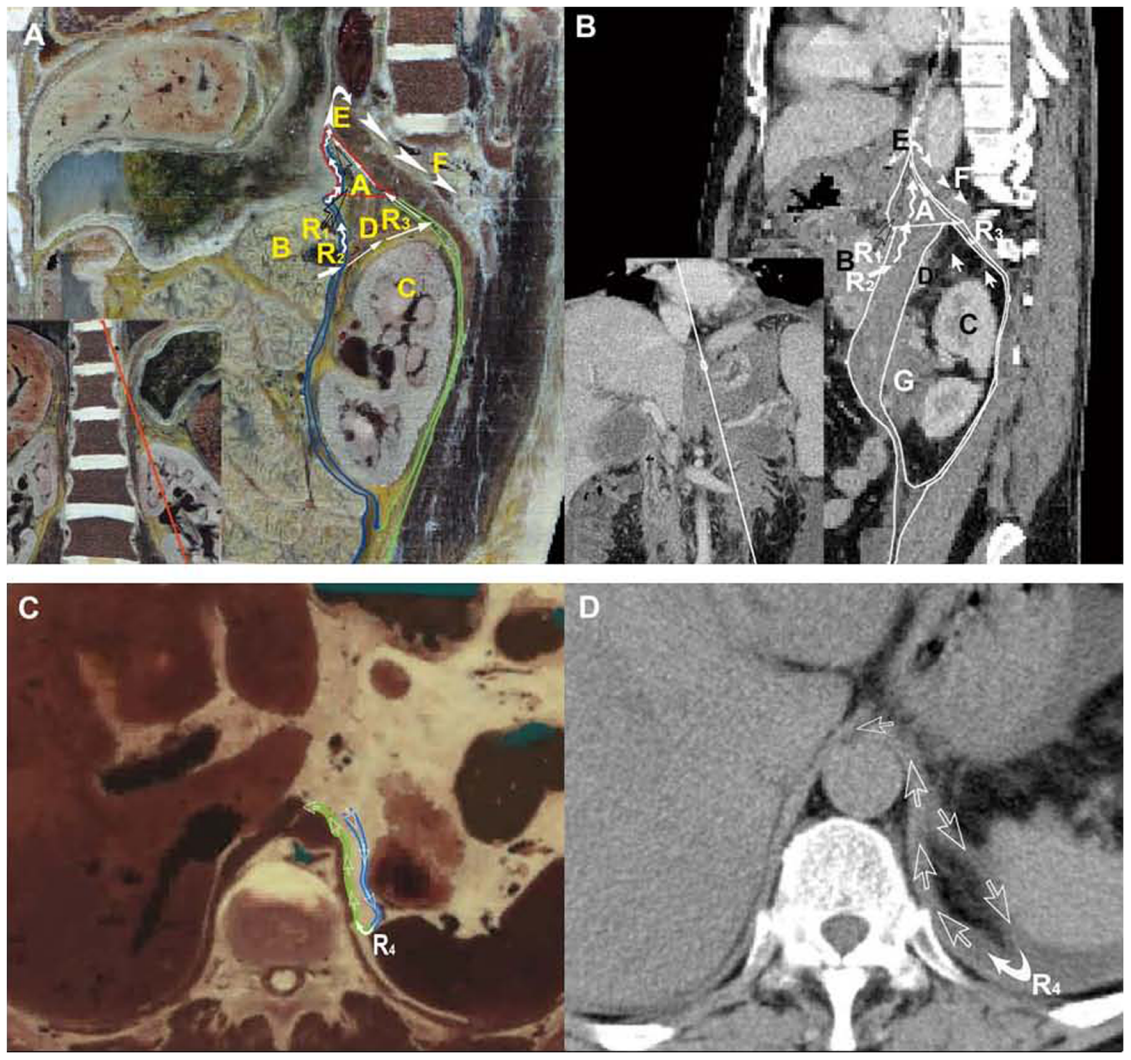
Visualization of pathways into the retrocrural space across the esophageal hiatus. (**A**) The oblique sagittal plane shows Route 1, Route 2 and Route 3 track together from the left extraperitoneal space to the retrocrural space across the esophageal hiatus (white arrowheads) from the lateral superior view on CVH2. Route 1 (black open arrows) is displayed as the path from the peripancreatic space to the left extraperitoneal space. Route 2 (crooked arrows) presents the conduit in the retromesenteric plane (blue lines) spreading to the left extraperitoneal space. Route 3 (white long arrows) shows the pathway from the retromesenteric plane to the retrorenal plane (green lines) and further to the left extraperitoneal space. (**B**) Case 1, a 47-year-old man with recurrent acute pancreatitis. Route 1 to Route 3 on the reformed CT plane corresponds well to the paths on CVH2. The thickened bridging septa distributes on Route 3 (white short arrows). A: left extraperitoneal space; B: pancreas; C: left kidney; D: left adrenal gland; E: esophageal hiatus; F: retrocrural space; G: pericapsular fluid collections. (**C**) Route 4 (white open arrows) from the retromesenteric plane to the left retrorenal plane across the left fascial trifurcation and further into the retrocrural space via the esophageal hiatus is displayed on the dataset of VHPA. (**D**) Case 2, a 60-year-old woman with recurrent acute pancreatitis. Route 4 is present in the CT image.

**Figure 2:**
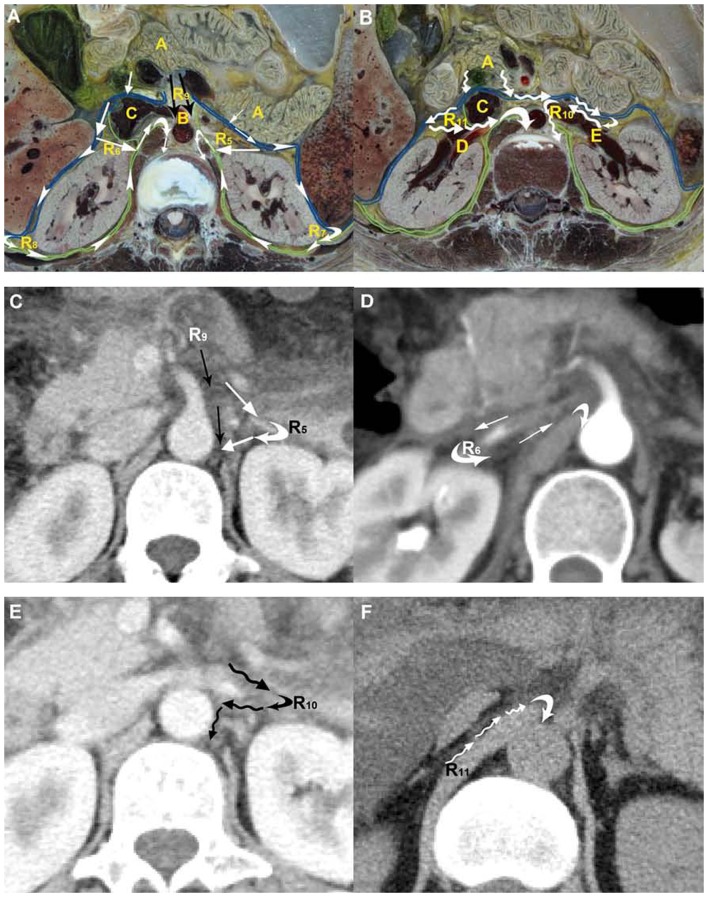
Manifestation of pathways into the retrocrural space across the aortic hiatus. (**A, B**) Route 5 to Route 11 into the retrocrural space across the aortic hiatus is displayed on CVH2. (**A**) The potential Route 5 to Route 9 is shown in the superior mesenteric artery section. Route 5 (white arrows) is from the retromesenteric plane to the left retrorenal plane via the left bridging septa, and further into the retrocrural space across the aortic hiatus; Route 6 is present as the similar conduit on the opposite side. Route 7 (arrowheads) is from the retromesenteric plane to the left retrorenal plane through the left fascial trifurcation and additionally into the retrocrural space; Route 8 is the contralateral pathway into the retrocrural space. Route 9 (black arrows) is the channel into the retrocrural space along the superior mesenteric artery. (**B**) Route 10 and Route 11 (crooked arrows) are the pathways along the left renal vein and right renal artery into the retrocrural space across the aortic hiatus on the left renal vein section. A: pancreas; B: superior mesenteric artery; C: inferior vena cava; D: right renal artery; E: left renal vein. (**C, E**) Case 1, a 34-year-old woman with recurrent acute pancreatitis. Route 5, Route 9 and Route 10 are present as fluid spreading along the left bridging septa (white arrows), superior mesenteric artery (black arrows) and left renal vein (crooked arrows) into the retrocrural space, respectively. (**D**) Case 2, a 47-year-old woman with recurrent acute pancreatitis. Route 6 is displayed as the fluid extending to the retrocrural space via the right bridging septa (white arrows). (**F**) Case 3, a 42-year-old man with recurrent acute pancreatitis. Route 11 is manifested as the fluid flowing along the right renal vein (crooked arrows) to the retrocrural space across the aortic hiatus.

**Figure 3:**
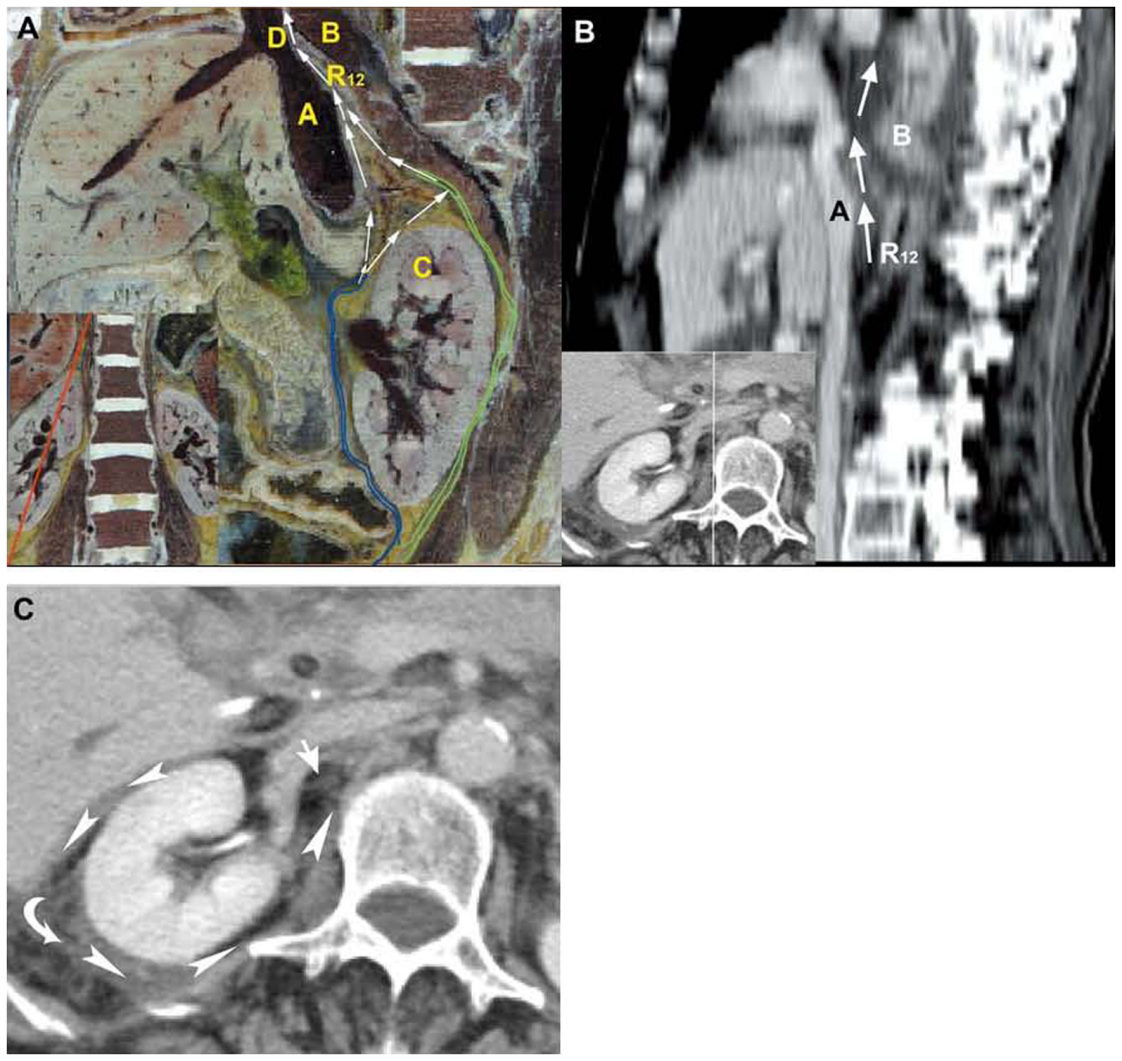
Presentation of the pathways into the right mediastinum via the inferior vena cava hiatus. (**A**) The oblique sagittal plane shows the potential, cranial Route 12 (long arrow) from the retromesenteric plane (blue lines) and retrorenal plane (green lines) to the bare area of the liver, and further into the right mediastinum via the inferior vena cava hiatus from the medial inferior view on CVH2. (**B, C**) A 72-year-old woman with recurrent acute pancreatitis. Sagittal plane shows Route 12, in which the fluid drains from the bare area of the liver to the right mediastinum across the inferior vena cava hiatus (long arrow). The peripancreatic fluid in the mediastinum further develops to the pleural effusion and the right lower lobar lung consolidation. The axial CT image depicts the pancreatic fluid flows from the retromesenteric plane to the right retrorenal plane through the right fascial trifurcation (arrowheads) and the bridging septa (short arrow). A: inferior vena cava, B: right lung, C: right kidney, D: inferior vena cava hiatus.

**Figure 4:**
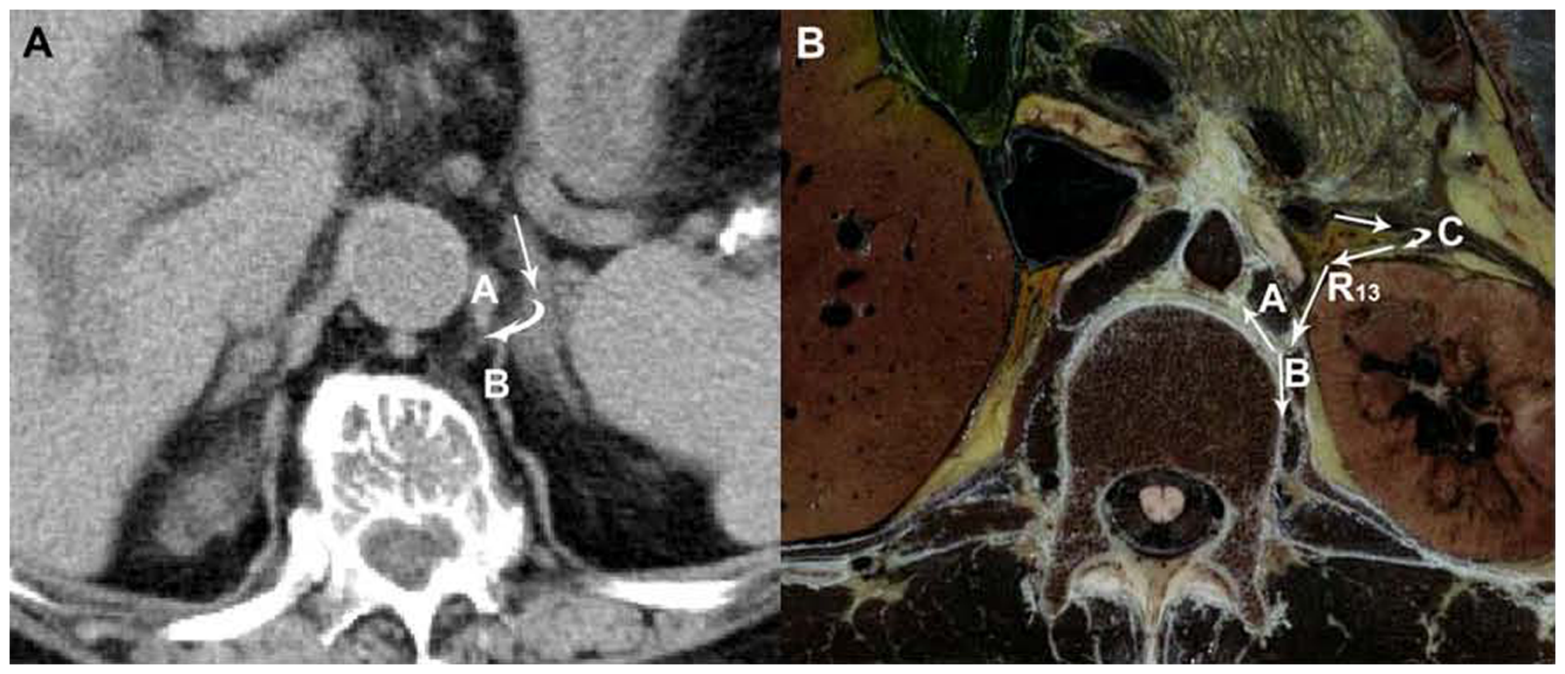
Visualization of the pathway into the retrocrural space by penetrating the diaphragm. (**A**) A 89-year-old woman with recurrent acute pancreatitis. The CT image manifests Route 13, in which the pancreatic fluid extends from the left perirenal space to the retrocrural space via the narrow space on the left diaphragm (white arrow). (**B**) The axial CVH5 image illustrates the potential pathway of Route 13 from the left perirenal space to the retrocrural space through the narrow space between the left costal diaphragm and left crural diaphragm. A: crural diaphragm, B: costal diaphragm, C: retromesenteric plane.

Generally, most of the fluid drains into the mediastinum via the esophageal hiatus, aortic hiatus and inferior vena cava hiatus by way of four kinds of routes, five kinds of routes and one kind of route, respectively (Figs 1, 2 and 3). In addition, the fluid could spread into the retrocrural space by penetrating the left diaphragm (Fig. 4).

### 1 Pathways into the retrocrural space across the esophageal hiatus

Route 1 to Route 4 served as four potential routes spreading into the retrocrural space via the esophageal hiatus in Visible Human Project images (Fig. 1). Route 1 was from the peripancreatic space to the left extraperitoneal space directly and additionally into the retrocrural space via the esophageal hiatus (Fig. 1A, B). Route 2 was from the peripancreatic space to the left extraperitoneal space via the retromesenteric plane, and further into the retrocrural space via the esophageal hiatus (Fig. 1A, B). If the latent pathway from the peripancreatic space into the retromesenteric plane had formed, Route 3 and Route 4 went from the retromesenteric plane to left retrorenal plane along the left perirenal bridging septa or across the left fascial trifurcation, and further tracked up into the retrocrural space by way of the left extraperitoneal space and esophageal hiatus (Fig. 1). Corresponding to these four routes in Visible Human images, CT scans showed that Route 1 and Route 2 were present in 66.7% (34/51) and 70.6% (34/51) of the patients, respectively. The appearance rates of Route 1 and Route 2 were 27.4% (34/124) and 29.0% (36/124) in all pathways, respectively. What’s more, the occurrence frequency of both Route 3 and Route 4 in patients by way of the left retrorenal plane was 17.7% (22/124) for the entire drainage system.

### 2 Pathways into the retrocrural space across the aortic hiatus

Figures 2A and 2B illustrate Route 5 to Route 11, respectively, as the potential paths of fluid spread by way of the interfascial planes and/or the perivascular space from the peripancreatic space to the retrocrural space across the aortic hiatus on CVH2 images. Route 5 was the pathway from the retromesenteric plane to the left retrorenal plane across the left bridging septa, and further to the retrocrural space via the aortic hiatus (Fig. 2A, C). In contrast, Route 6 was displayed as the conduit from the retromesenteric plane to the retrocrural space through a similar right pathway (Fig. 2A, D). The channel from the retromesenteric plane to the left retrorenal plane across the left fascial trifurcation, and then further to the retrocrural space via the aortic hiatus, was Route 7 (Fig. 2A). The analogous right pathway was Route 8 (Fig. 2A). The pathway along the celiac trunk and/or the superior mesenteric artery into the retrocrural space via the aortic hiatus was Route 9 (Fig. 2A, C). Route 10 was manifested as the pathway from the retromesenteric plane to the left perirenal space, and then along the left renal artery or vein to the retrocrural space across the aortic hiatus (Fig. 2B, E). Similarly, Route 11 was the conduit from the retromesenteric plane to the retrocrural space through the contralateral, corresponding perivascular pathway (Fig. 2B, F). Corresponding to the pathways on Visible Human Project images, the CT images of 39.2% (20/51) of the patients with an involved retrocrural space clearly displayed that the fluid extended from the peripancreatic space to the retrocrural space across the aortic hiatus; the fluid drained across Route 5, Route 6, Route 9, Route 10 and Route 11 in eight, two, six, three and five patients, respectively. The appearance rate of the drainage routes to the retrocrural space through the aortic hiatus was 19.4% (24/124) in all pathways. Route 7 and Route 8 were not displayed in any CT images.

### 3 Pathways into the right posterior mediastinum via the inferior vena cava hiatus

Route 12 was displayed as a pathway from the fascial planes to the bare area of the liver cranially and then further into the mediastinum across the inferior vena cava hiatus in Visible Human Project images (Fig. 3). CT scans showed that Route 12 was present in only one patient.

### 4 Pathways into the retrocrural space by penetrating the left diaphragm

As we explored routes via diaphragmatic hiatuses into the mediastinum in CT images, we found another route in which the fluid extended from the peripancreatic space to the left perirenal space, which then intruded into the retrocrural space via the narrow space on the left diaphragm (Fig. 4A). This path was defined as Route 13 and was manifested in 13.7% (7/51) of the patients. The occurrence rate of Route 13 was 5.6% (7/124) for all pathways. After looking at the Visible Human Project images, the narrow space was confirmed to be located between the left costal diaphragm and the left crural diaphragm (Fig. 4B).

## Discussion

The achievements of this study have set up a new pattern for exploring the communication relationships of the retroperitoneal space. Firstly, we analyzed potential communication relationships among these spaces in the retroperitoneal space in Visible Human Project images based on the concept of fascial planes. Secondly, by using a good indictor for communication relationships of the retroperitoneal space, the retroperitoneal extension of the pancreatic fluid complacently confirmed the distribution of fascial planes and the rich network of bridging septa, and the drainage routes in CT images. Moreover, the objective drainage pathways in CT images can correct some false hypotheses about latent conduits obtained in Visible Human Project images. Lastly, additional routes that have not been considered in Visible Human Project images can be displayed in CT images.

In our study about pathways from the peripancreatic space to the mediastinum, secondary inflammatory fibrosis from previous acute pancreatitis prevented fluid from draining along traditional advantageous routes, which allowed the mediastinal extension of pancreatic fluid to occur. In addition, we believe the pumping mechanism of breathing plays a pivotal role in this course. The involved frequency of Route 1 (27.4%) and Route 2 (29.0%) was obviously higher than that of other pathways into the mediastinum. As the two most important conduits to the mediastinum, clinicians should attach more importance to Route 1 and Route 2.

**Figure 5:**
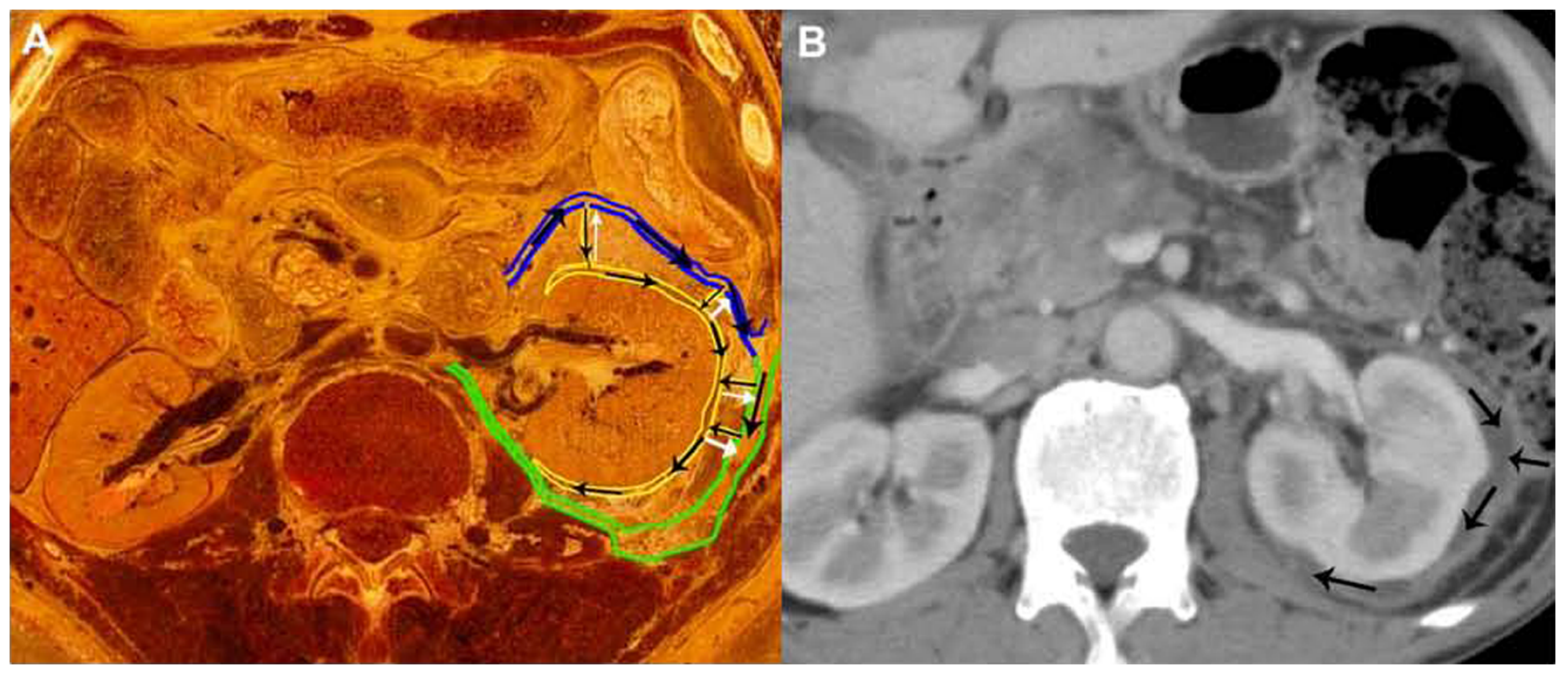
Displayed conduits across the left bridging septa to the subcapsular space. (**A**) The left perirenal bridging septa may provide a latent channel for renal subcapsular fluid to spread to the fascial planes (white arrows). Conversely, these pathways may be recruited to drain pancreatic fluid from the fascial planes to the subcapsular space in acute pancreatitis (black arrows). (**B**) A 61-year-old man with carcinoma at the pancreatic head, which led to secondary pancreatitis. The pancreatic fluid extends along the left bridging septa to the subcapsular space (black arrows).

During the research, we found Route 7 and Route 8 were not displayed in any CT images. Perhaps it is too hard to open all the fascial planes along the retromesnteric plane, bilateral fascial trifurcation and bilateral retrorenal plane because of secondary inflammatory fibrosis from previous acute pancreatitis. Also, the left perirenal bridging septa may provide a conduit for rapidly developing renal subcapsular hematoma to reach the expansile fascial planes [8], [10]. On the contrary, we argue that this type of pathway can be recruited to drain pancreatic fluid from the fascial planes to the subcapsular space for decompression (Fig. 5A). Compared to the pancreas lying before the left perirenal space, the pancreatic head, the uncinate process, the descending duodenum, and even the inferior vena cava are located before the right perirenal space, and the inferior hepatic surface under the right lobe may compress the above structures in a backward manner (Fig. 5). These anatomic characteristics make the right interfascial planes to be stressed, so the right conduit to the retrocrural space across Route 8 cannot be formed. Scialpi reported that the right perirenal space was closed medially [8]. However, we found the fluid in the bilateral perirenal space could extend along the renal vessels (Route 10 and Route 11) to the mediastinum, which suggests the right perirenal space is open medially on renal vessel sections (Fig. 2F).

Route 13, in which the fluid collections spread into the retrocrural space across the left narrow space between the left costal diaphragm and crural diaphragm, was initially proposed (Fig. 4) [23]. Since the pancreatic head, the uncinate process and the descending duodenum lead to straightness of the medial part of the right perirenal space, the right adrenal gland is adjacent to the right narrow space corresponding to the left space. A draining path similar to Route 13 cannot be constructed lightly on the right side.

Accompanied by chronic development of acute pancreatitis, the above 11 routes have the potential to transform to a fistulous tract into the mediastinum. A timely and accurate diagnosis is important for potentially life-threatening presentation of mediastinal pseudocysts and pancreaticopleural fistulas [17]. If the detailed anatomy of drainage routes to the mediastinum becomes well known using the platform of computer-assisted medicine to display on Visible Human Project images compared with CT images in real-time from patients with acute pancreatitis, the diagnostic accuracy of diseases will be increased. This will provide radiologists with realistic normal controlled views, which should facilitate correct interpretation of CT images of these two entities. In addition, because the definitive treatment for these two entities is still evolving, familiarity with drainage routes may contribute to the development of new treatment methods.

Our study has several limitations. Firstly, there was a small number of patients in the study. We are planning to conduct a large multicenter study to confirm the drainage pathways into the mediastinum. Secondly, we should study some cases of mediastinal pseudocysts and pancreaticopleural fistulas to observe realistic cases of fistulas into the mediastinum in CT images and analyze whether there are differences between the routes to the mediastinum in recurrent acute pancreatitis and fistularization in the above two diseases. Last, we should introduce plastination technology into research about conduits that are distributed in fascial planes and should compare this imaging modality with our research method [24].

In conclusion, our study has established a new pattern for exploring the communication relationships in the retroperitoneal space. This kind of exploration model for imaging anatomy may be used to study the communication relationships in the abdominal spaces, mediastinal spaces, cervical fascial spaces and cisterns in the skull. Mastering these relationships may be beneficial for clinicians to study the spread of bacteria and inflammation in these regions. Familiarity with the entire anatomy of drainage pathways to the mediastinum may be helpful for increasing the diagnostic accuracy of mediastinal pseudocysts and pancreaticopleural fistulas in acute pancreatitis.
